# Silencing the noise: APOC3 inhibition and the long-overdue reckoning with triglycerides

**DOI:** 10.1016/j.ajpc.2026.101551

**Published:** 2026-03-24

**Authors:** Michael D. Shapiro, Khuram Nasir

**Affiliations:** Department of Cardiovascular Medicine, Center for Prevention of Cardiovascular Disease, Wake Forest University School of Medicine, Medical Center Boulevard, Winston Salem, NC 27157, United States

**Keywords:** Apolipoprotein C-III (APOC3), Hypertriglyceridemia, Triglyceride-rich lipoproteins, Remnant cholesterol

For decades, we have watched the triglyceride story unfold with a kind of weary ambivalence. The observational epidemiology has been consistent. Elevated triglycerides track with atherosclerotic risk in population cohorts worldwide. Yet, there is a graveyard of failed triglyceride lowering therapies, at least from the perspective of cardiovascular outcomes. The disappointment in these approaches has led many clinicians to question whether triglyceride lowering per se is a viable cardiovascular therapeutic strategy. However, this apparent failure may actually reflect the limitations of prior drug classes rather than any fundamental flaw in the underlying biology. The emergence of RNA-based therapeutics targeting apolipoprotein C-III (APOC3) represents a mechanistically distinct and potentially transformative approach to this problem, one grounded in robust human genetic validation that was absent for prior triglyceride -lowering strategies.

## 1 APOC3 as a validated therapeutic target

APOC3 is synthesized predominantly in the liver and exerts its hypertriglyceridemic effect through multiple mechanisms: inhibition of lipoprotein lipase (LPL)-mediated TRL hydrolysis, direct impairment of hepatic TRL clearance via LPL-independent pathways, and interference with apolipoprotein E–mediated hepatic uptake [1]. Heterozygous and homozygous loss-of-function variants in *APOC3* are associated with markedly reduced circulating triglyceride levels, favorable non-triglyceride lipoprotein profiles, and reduced cardiovascular mortality in the absence of adverse clinical phenotypes [2,3]. These observations closely resemble the PCSK9 saga, a story that presaged the development of highly effective LDL-C–lowering therapies. Mendelian randomization analyses further confirm the causal role of APOC3 in both ASCVD risk and acute pancreatitis (AP) risk, establishing a principled basis for therapeutic APOC3 inhibition [4].

Two mechanistically distinct RNA-based therapeutics have now been developed against this target. Olezarsen is a GalNAc-conjugated antisense oligonucleotide (ASO) that degrades *APOC3* mRNA through RNase H–mediated cleavage. Plozasiran is a GalNAc-conjugated small interfering RNA (siRNA) that achieves target silencing through the RNA-induced silencing complex (RISC). Both confer hepatocyte selectivity through GalNAc–asialoglycoprotein receptor interactions, and both have received FDA approval for reduction of triglycerides in adults with familial chylomicronemia syndrome (FCS). However, mechanistic differences between ASOs and siRNAs, including their intracellular localization, duration of activity, and downstream cellular effects, may produce clinically meaningful differences in off-target biology, particularly with respect to hepatic lipid metabolism and glycemic regulation.

## 2 What the data show

In patients with severe hypertriglyceridemia (SHASTA-2), plozasiran 25 mg administered subcutaneously every three months produced median triglyceride reductions of 82% and 83% at months 12 and 24 of the open-label extension, respectively [5]. These dramatic reductions in triglycerides persisted over the course of the trials. Patients who had been on placebo during the randomized phase and then crossed over achieved reductions indistinguishable from those who had received active drug all along, an important observation suggesting no meaningful attenuation of effect over time and no immunogenic tachyphylaxis that one might worry about with biological agents.

In the mixed hyperlipidemia population of MUIR, patients typical of those seen commonly in the clinic, with triglycerides in the 150–499 mg/dL range and elevated non-HDL-C, median reductions in triglycerides were 67% at 12 and 24 months [5]. The accompanying lipoprotein changes were broadly favorable. Substantial reductions in remnant cholesterol (71% in SHASTA-2 at month 24, 56% in MUIR), meaningful non-HDL-C reductions, apoB reductions in the 7–19% range depending on the population, and HDL-C increases of 60–85% were observed. In the MUIR population, LDL-C actually fell by a median of 22%, a pattern consistent with the clearance of cholesterol-rich remnant particles. Across both studies, no AP events were adjudicated during the OLE period. In the SHASTA-2 randomized phase, acute pancreatitis occurred in 3.3% of placebo patients and 0.6% of plozasiran-treated patients within 12 months. The authors appropriately model a virtual placebo survival curve to contextualize this absence of events, and while such simulations have inherent limitations, the clinical logic is straightforward. A patient population carrying median triglycerides of 685 mg/dL at baseline, reduced to approximately 120 mg/dL sustained over two years, is simply not going to experience pancreatitis at the same rate.

Hypertriglyceridemia and type 2 diabetes are frequent companions, which makes the glucose signal associated with potent APOC3 inhibition clinically relevant. The mechanistic explanation that robust triglyceride hydrolysis liberates fatty acids that preferentially undergo oxidation, transiently increasing gluconeogenic substrate, is biologically plausible. Both approved APOC3-targeting therapies, olezarsen and plozasiran, carry labeling language around glucose, though the magnitude and clinical significance appear to differ.

In the Phase 3 program evaluating olezarsen, a prespecified hepatic MRI substudy was embedded within the CORE trials [6]. Olezarsen was associated with a dose dependent increase in liver fat fraction over 12 months compared with placebo. Although these increases were generally not accompanied by marked elevations in transaminases and did not translate into overt hepatic clinical events during the follow up period, they raise mechanistic questions about altered hepatic lipid trafficking with sustained APOC3 inhibition. In parallel, small increases in fasting glucose and HbA1c were observed in treated patients, and hyperglycemia was reported more frequently than with placebo in some analyses. The magnitude of dysglycemia appeared modest, but in a population already enriched for metabolic risk, even small shifts in glycemic parameters warrant attention. These findings underscore the need for longer term metabolic surveillance as APOC3 inhibition moves into broader clinical use.

In SHASTA-2, an exploratory MRI substudy found no change in hepatic fat, though this was much smaller than the imaging study with olezarsen [7]. Hyperglycemia was reported more frequently with plozasiran than with placebo, and small mean increases in fasting glucose and HbA1c were observed over time, particularly among individuals with preexisting diabetes or prediabetes. The absolute changes in glycemic indices were modest. It is likely that with attention to diet, weight management, and glycemic pharmacotherapy, this is a manageable signal, not a disqualifying one.

## 3 The road ahead

The genetic modeling data cited by the authors are worth reflecting on. A reduction of remnant cholesterol by 13–32 mg/dL is projected to yield approximately 20% reductions in recurrent major adverse cardiovascular events [8]. In the MUIR OLE, mean remnant cholesterol fell from 47 mg/dL at baseline to roughly 20 mg/dL by month 24, an absolute reduction well within that modeled range. We do not yet have an outcomes trial for ApoCIII in this population. However, if the causal signal truly reflects the burden of triglyceride rich apoB containing lipoproteins rather than triglyceride mass per se, then sustained ApoCIII inhibition in patients with moderate hypertriglyceridemia may represent a biologically coherent strategy for event reduction. The common patient with moderate hypertriglyceridemia remains undertreated and overrepresented in residual risk cohorts despite statin therapy. A well designed, adequately powered cardiovascular outcomes trial in this group would test whether translating the genetic and mechanistic promise of ApoCIII inhibition into durable reductions in remnant cholesterol can meaningfully bend ASCVD event curves beyond contemporary standard of care.

For now, what these data establish is clear. Plozasiran durably silences APOC3, comprehensively remodels the atherogenic lipoprotein profile in both the severe and moderate hypertriglyceridemia settings, substantially reduces pancreatitis events over two years of follow-up, with a safety profile that appears manageable. The pharmacology is elegant and the quarterly dosing is practical. Most importantly, the strategy is anchored in human loss-of-function genetic validation. Preventive cardiologists have long focused on LDL-C while residual risk persisted in patients with atherogenic dyslipidemia. APOC3 inhibition now presents a biologically coherent opportunity to address that risk. Its ultimate place in preventive cardiology, however, will depend on definitive ASCVD outcomes trials. What the genetics promised, and what these data now make plausible, deserves to be tested in the patients who need it most.

## CRediT authorship contribution statement

**Michael D. Shapiro:** Conceptualization, Project administration, Writing – original draft. **Khuram Nasir:** Writing – review & editing.

## Declaration of competing interest

The authors declare the following financial interests/personal relationships which may be considered as potential competing interests: Michael D. Shapiro reports a relationship with Novartis that includes: consulting or advisory. Michael D. Shapiro reports a relationship with Amgen Inc that includes: consulting or advisory. Michael D. Shapiro reports a relationship with Merck & Co Inc that includes: consulting or advisory. Michael D. Shapiro reports a relationship with Alnylam Pharmaceuticals Inc that includes: consulting or advisory. Michael D. Shapiro reports a relationship with AstraZeneca that includes: consulting or advisory. If there are other authors, they declare that they have no known competing financial interests or personal relationships that could have appeared to influence the work reported in this paper.
